# A bibliometric analysis of the research landscape of vulvar cancer

**DOI:** 10.1007/s12672-026-04573-5

**Published:** 2026-02-17

**Authors:** Gilbert Georg Klamminger, Meletios P. Nigdelis, Mathias Wagner, Annette Hasenburg, Yaman Degirmenci

**Affiliations:** 1 Department of Obstetrics and Gynecology, University Medical Center of the Johannes Gutenberg University Mainz, Langenbeckstraße 1, 55131 Mainz, Germany; 2 Department of Gynecology, Obstetrics and Reproductive Medicine, Saarland University Medical Center (UKS), 66424 Homburg, Germany; 3 Department of General and Special Pathology, Saarland University (USAAR) and Saarland University Medical Center (UKS), 66424 Homburg, Germany

**Keywords:** Vulvar cancer, Bibliometrics, Scientometrics, Web of Science

## Abstract

**Background:**

We present a comprehensive bibliometric analysis to characterize the contemporary research landscape of vulvar cancer (VC).

**Materials and methods:**

To identify publications relevant to VC research, a systematic search of the Web of Science (WoS) database was conducted. Key bibliographic information was extracted, and quantitative analyses and network mapping (VOSviewer) were performed.

**Results:**

A total of 3702 publications were identified, with the earliest record dating back to 1946 and a steadily increasing publication volume over recent decades. Most of the literature has been published in English. The leading journals publishing VC research were *Gynecologic Oncology* (Elsevier) and the *International Journal of Gynecological Cancer* (Elsevier). A subsequent keyword analysis revealed distinct conceptual domains within the field, and an examination of the ten most frequently cited articles highlighted recurrent clinically relevant themes, including lymph node assessment/management and HPV genotype distribution.

**Conclusion:**

In this analysis, we outline key research trends, influential regions, leading journals, and the most cited works in VC research. Such bibliometric summaries may serve as a useful guide for future research efforts and support funding decisions.

**Supplementary Information:**

The online version contains supplementary material available at 10.1007/s12672-026-04573-5.

## Introduction

Despite a pronounced regional variation in the incidence of vulvar cancer (VC), with particularly high age-standardized incidence rates reported in Germany (4.2 per 10,000) and South Africa (7.2 per 10,000), the global incidence is increasing [1]. Research efforts and the standardization of clinical workflows remain underdeveloped, which is reflected in the marked heterogeneity observed across diagnostic and therapeutic recommendations in international guidelines [2] – a limitation that can be partly attributed to factors such as the predominance of studies with small sample sizes and the scarcity of data on precision medicine approaches [3, 4]. Moving forward, these challenges highlight the necessity of internationally coordinated initiatives and the critical need for expanded translational research and a systematic and structural assessment of the current research landscape. Owing to its diverse pathobiology (HPV-associated and HPV-independent carcinogenesis), high morbidity, substantial impact on quality of life [5, 6], and the need for interdisciplinary treatment approaches, VC intersects with key themes in modern cancer research and should be regarded as highly relevant in contemporary oncology.

By analyzing citation-based publication patterns, bibliometric methods offer a valuable approach for elucidating key trends in clinical practice and understanding the evolution of research behavior over time [7–10]. To the best of our knowledge, no dedicated bibliometric analysis has been performed in the field of VC research. Consequently, comprehensive insights into publication output, contributing countries, journals, and frequently used keywords are lacking. In this brief report, we aim to address this specific gap in the literature by (a) quantifying the volume of VC research, (b) characterizing the types of publications produced, and (c) assessing the contributions of individual countries and their collaborative networks to VC research. Overall, this short summary may help support and optimize future strategic research planning.

## Materials and methods

Data and corresponding metrics were retrieved from the Web of Science (WoS) database on 7. November 2025, employing a set of predefined search terms and Boolean operators (TS=(“vulvar cancer” OR “vulvar carcinoma” OR “vulva neoplasm” OR “vulva neoplasms”)). The WoS database was employed in this study because it offers extensive multidisciplinary coverage, integrates well-established citation tracking functionalities, and applies rigorous inclusion standards for indexed journals. The search applied no restrictions on publication date, language, journal, or country of origin. Information on authors’ full names, article titles, source titles, languages, document types, keywords, addresses, affiliations, citation counts, cited reference counts, publisher details, publication year, DOI/ISSN/eISSN/PubMed ID, and WoS categories was extracted both into Microsoft Excel (Version 16.78, 2023) (allowing for initial quantitative data analysis after detection of duplicates via conditional formatting and manual deletion of duplicate titles) and to the advanced bibliometric analysis platform VOSviewer (version 1.6.20, Ⓒ Nees Jan van Eck and Ludo Waltman). The latter allows for a comprehensive, multidimensional examination of the extracted articles by creating and displaying network structures within the bibliographic data (keyword co-occurrence analysis, mapping of research collaborations across geographical areas and institutions, and identification of leading journals). Here, nodes are scaled according to publication volume/keyword impact, and the connecting lines visualize relationships that structure the network [11–13]. For the keyword analysis, threshold selection was guided by the overall number of publications. Accordingly, the minimum keyword occurrence threshold was manually set at 15 to reduce noise while maintaining an adequate coverage of niche themes. The study protocol was performed in alignment with the protocol (*STAR Protocols*) for conducting bibliometric analysis in biomedicine previously published by Qiang Du et al. [14]. All analyses were conducted in an exploratory manner. For a visual overview of the workflow, see Supp. Figure 1.

In the final step, the articles were sorted in descending order according to the individual citation count and assessed according to their title, keywords, and document type. Using abstract and full-text screening (if needed), the authors identified the 10 most highly cited and impactful clinical studies/research articles on vulvar neoplasms. Narrative literature reviews, statements, congress articles, and original articles not directly relevant to VC (e.g., basic science articles) were excluded from this sub-analysis, and citation densities for each article were calculated (number of citations/publication age in years).

## Results

In total, 3702 documents were extracted from the WoS core collection. Following the removal of explicit duplicates, particularly those arising from congress-related publications, 3673 articles remained eligible for quantitative analysis, spanning 1946 to 2025. The earliest identified publication on this topic, authored by Watson et al., was published in 1946 in the *American Journal of Obstetrics and Gynecology* (Elsevier) and delineated the symptoms and treatment of 30 patients with VC [15].

**Fig. 1:**
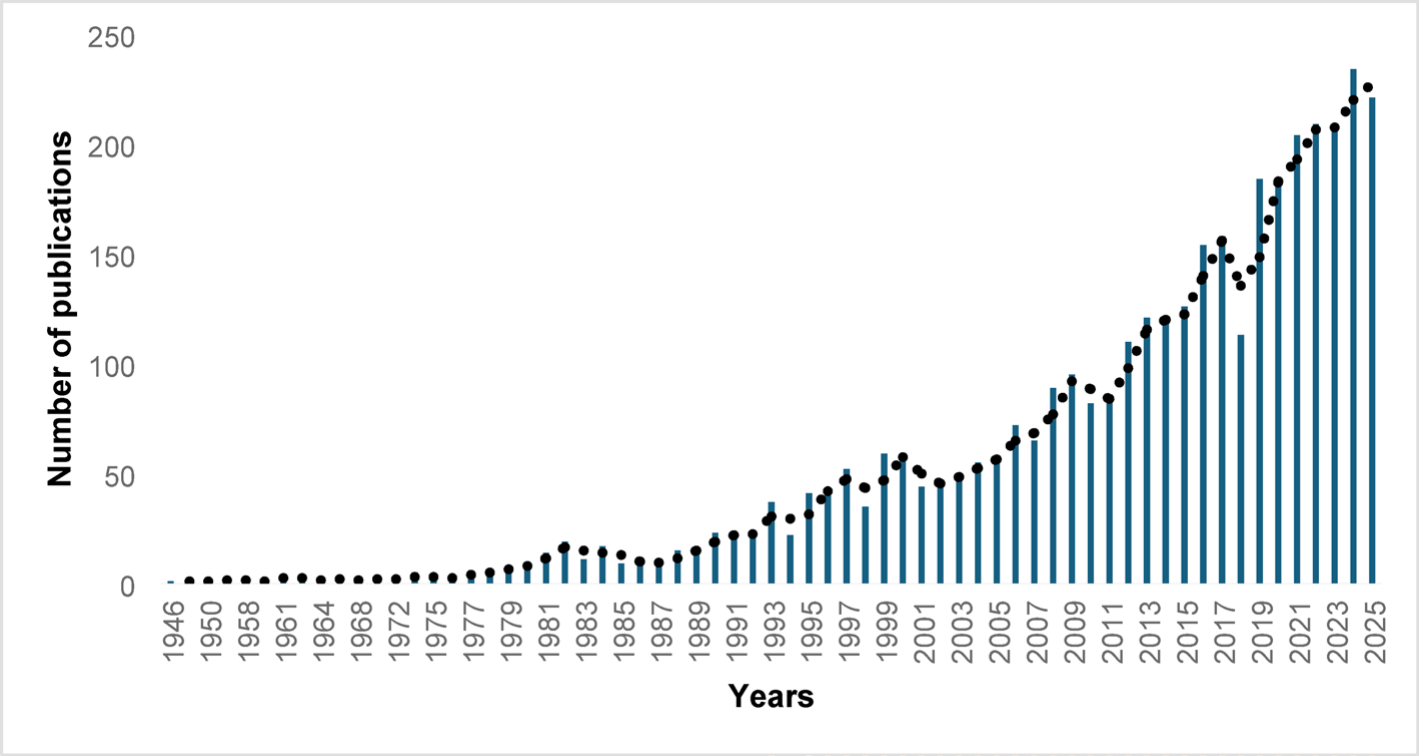
Annual scholarly VC research output from 1946 to 2025. X-axis: years; Y-axis: number of publications

Figure 1 presents the annual distribution of publications (publication volume), illustrating a steady rise in the number of published documents, accompanied by a parallel increase in the number of original articles (Supp. Figure 2). Correspondingly, the number of total citations per year (citation trends) is visualized in Supp. Figure 3.

With a total number of 2218 documents, the original article is the most common type of published document, followed by meeting abstracts (672 documents) and reviews (451 documents); see Supp. Table 1. Most publications were written in English (3469 documents), with 133 publications in German and 32 in French. Of the total publications, 503 documents were published in the journals *Gynecologic Oncology* (Elsevier) and I*nternational Journal of Gynecological Cancer* (*IJGC*, Elsevier). With a total of 15,064 citations (average citations per article: 29.9), *Gynecologic Oncology* had the most citations, followed by *Obstetrics and Gynecology* (4477 citations; average citations per article: 56.0) and *IJGC* (3944 citations; average citations per article: 7.8) (Table 1).

**Fig. 2:**
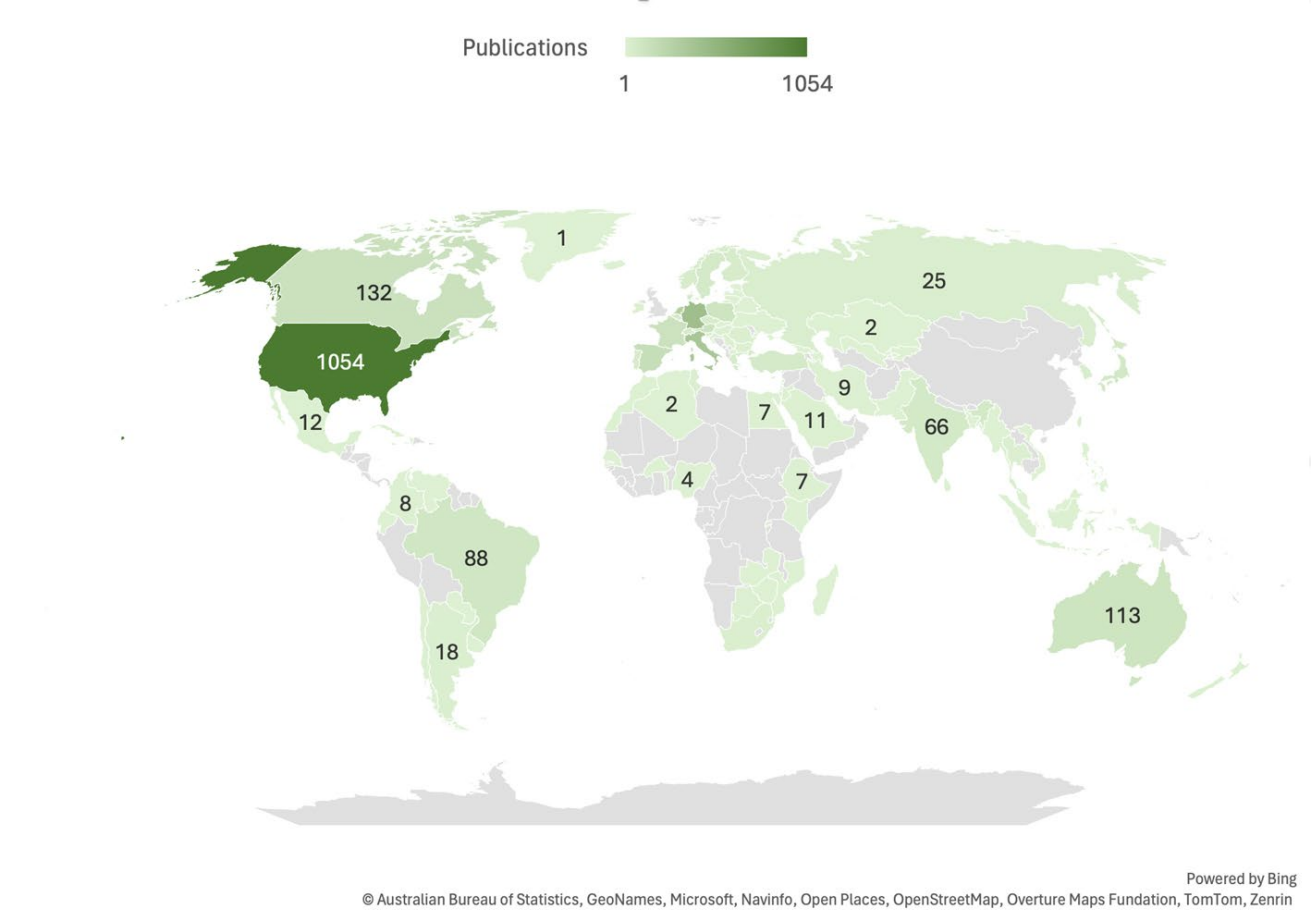
A filled map chart (constructed using Microsoft Excel, Version 16.78, 2023) illustrates the geographic distribution of VC research output; the numbers shown represent the total number of publications per country

**Fig. 3:**
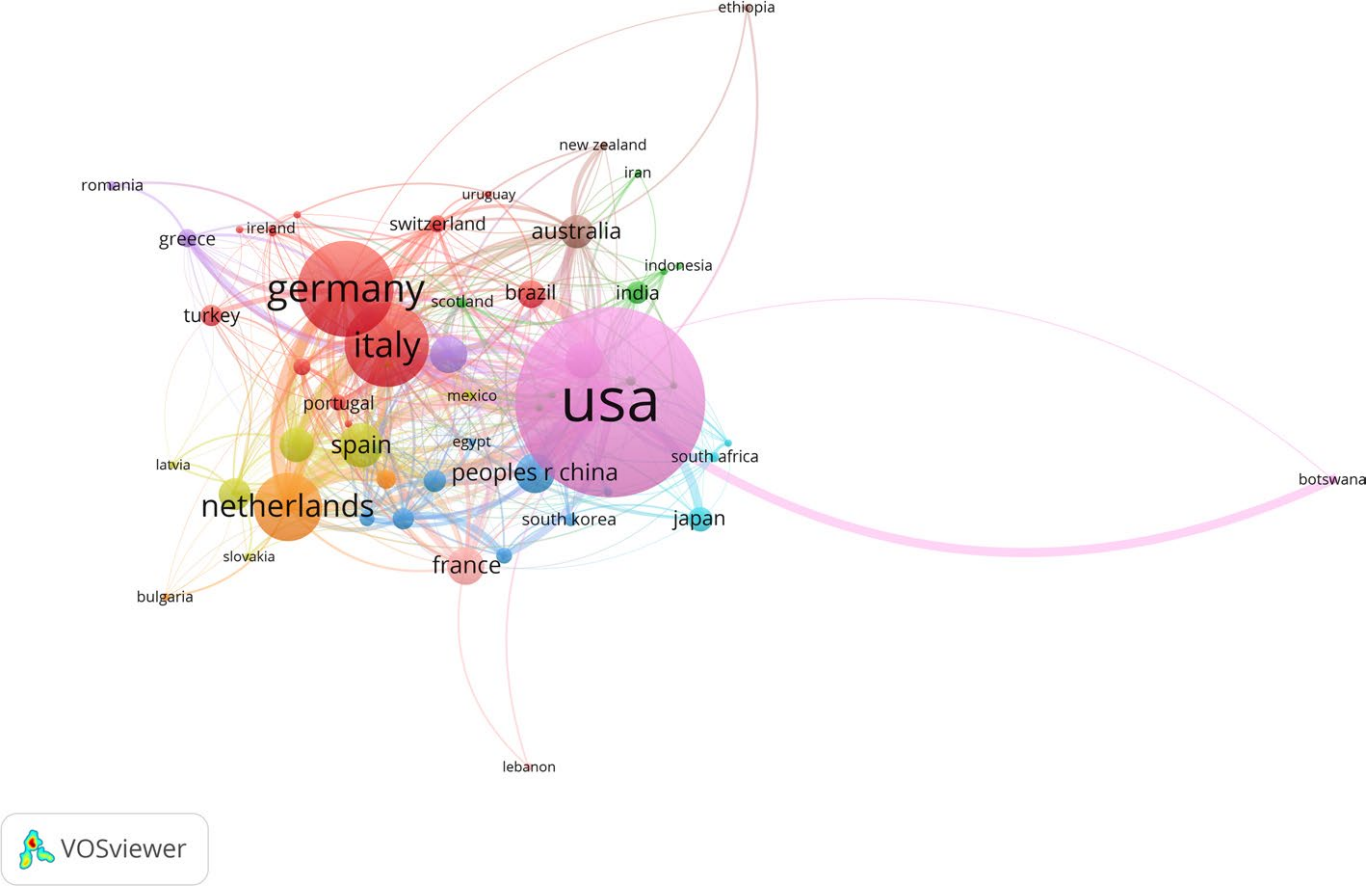
Collaborative network visualization of countries with display of their relations (co-authored documents) using VOSviewer software. The minimum number of documents per country is five; only countries connected via co-authorship are displayed

Examining the geographical distributions as well as the individual impact of single institutions within the domain of VC research, the United States leads the field by means of total publication volume (1054 documents) but also citations (in total: 31901; average citations per article: 30.3), followed by Germany (440 documents) and the Netherlands (10348 citations; average citations per article: 36.6), see Table 2; Fig. 2. The institutional analysis demonstrates *Università Cattolica Del Sacro Cuore* (Italy) as the leading most-publishing institution with 85 documents (1156 citations; average citations per article: 13.6) (Supp. Table 2). The relatedness of countries regarding the number of their co-authored documents (co-authorship analysis) is depicted as network visualization (Fig. 3)

**Table 1:** The ten most influential journals in VC research ranked by total publication output

Journal (Publisher)	Publication	Citations	Average citations per article
Gynecologic Oncology (Elsevier)	503	15,064	29.9
International Journal Of Gynecological Cancer (Elsevier)	503	3944	7.8
European Journal Of Gynaecological Oncology (MRE Press)	83	494	6.0
Obstetrics And Gynecology (Lippincott Williams & Wilkins)	80	4477	56.0
International Journal Of Radiation Oncology Biology Physics (Elsevier)	74	1252	16.9
Cancers (MDPI)	68	753	11.1
Geburtshilfe Und Frauenheilkunde (Thieme Medical Publishers)	60	115	1.9
Cancer (Wiley)	48	3859	80.4
Archives Of Gynecology And Obstetrics (Springer Nature)	47	487	10.4
American Journal Of Obstetrics And Gynecology (Elsevier)	44	1608	36.5

**Table 2:** The ten most productive countries in VC research, ranked by overall publication productivity

Country	Publications	Citations	Average citations per article
USA	1054	31,901	30.3
Germany	440	7313	16.6
Italy	370	8162	22.1
Netherlands	283	10,348	36.6
Spain	158	5414	34.3
Peoples R China	140	1337	9.6
Canada	132	5278	40.0
England	131	6790	51.8
France	128	5363	41.9
Austria	117	2941	25.1

**Fig. 4:**
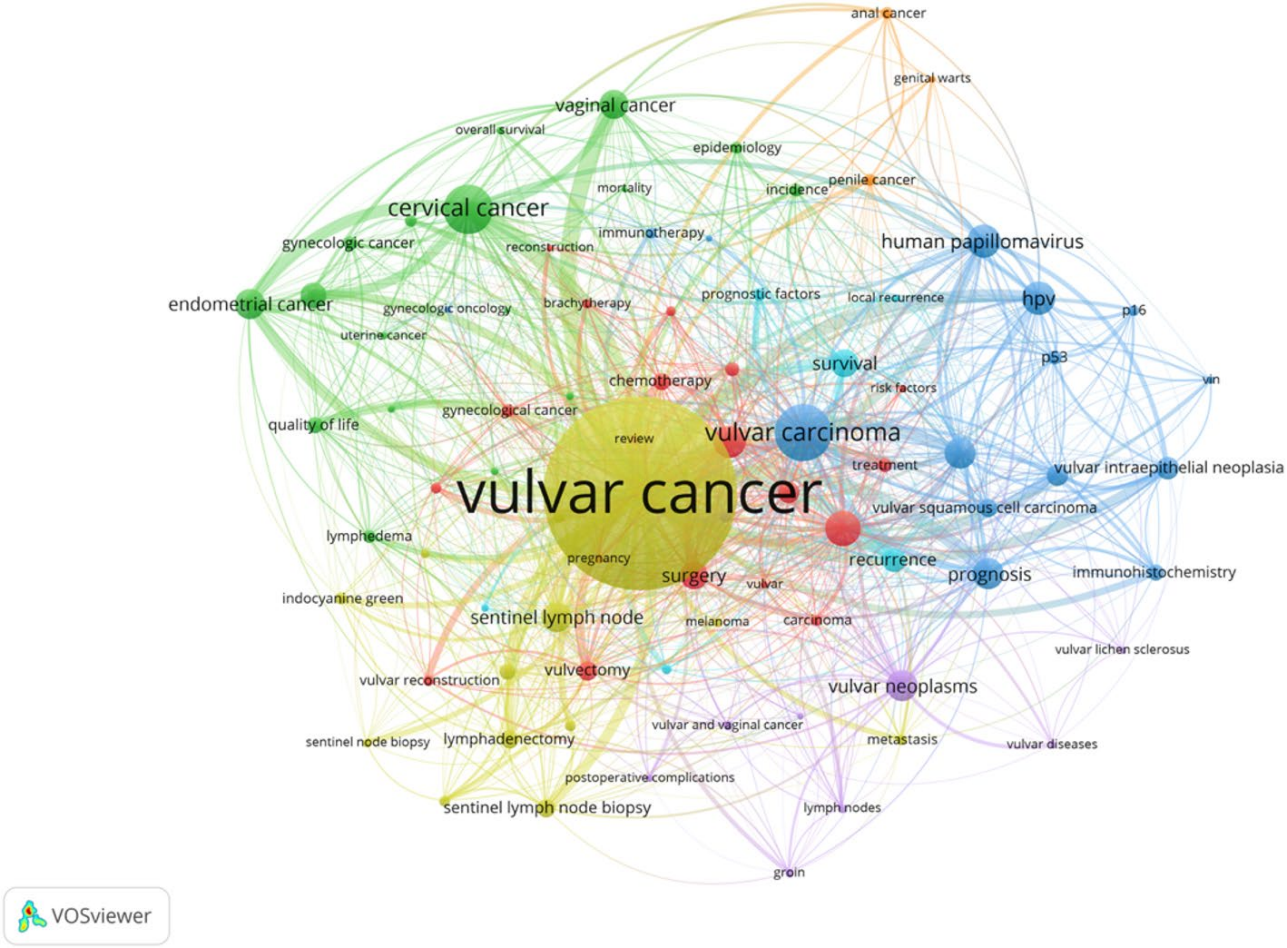
Network visualization of the most frequently used keywords in VC research using the VOSviewer software. Minimum keyword count: 15

The most common keyword employed was “Vulvar Cancer” (1141 hits), followed by “Vulvar Carcinoma” (234 hits), and “Cervical Cancer” (194 hits) (Fig. 4, Supp Table 3). The keyword analysis further delineated seven distinct sub-clusters (Table 3).

**Table 3:** A detailed presentation of the keyword analysis, organized into seven clusters along with the respective keywords within each cluster and the overall cluster topic

Keyword–number of clusters	Individual keywords	Thematic grouping/cluster topic
Cluster 1	brachytherapy, cancer, carcinoma, chemoradiation, chemotherapy, gynecological cancer, pregnancy, radiation therapy, radical vulvectomy, radiotherapy, reconstruction, review, risk factors, surgery, treatment, vulva, vulvar, vulvar reconstruction, vulvectomy	Therapy and treatment
Cluster 2	cervical cancer, complications, endometrial cancer, epidemiology, gynecological cancer, gynecologic malignancy, incidence, lymphedema, meta-analysis, morbidity, mortality, ovarian cancer, overall survival, pelvic exenteration, quality of life, uterine cancer, vaginal cancer	Epidemiology
Cluster 3	cervical carcinoma, gynecologic oncology, HPV, human papillomavirus, immunohistochemistry, immunotherapy, lichen sclerosus, p16, p53, prognosis, squamous cell carcinoma, VIN, vulvar carcinoma, vulvar intraepithelial neoplasia, vulvar squamous cell carcinoma	Tumor biology, pathology, and diagnosis
Cluster 4	indocyanine green, inguinal lymphadenectomy, inguinofemoral lymphadenectomy, lymphadenectomy, lymphoscintigraphy, melanoma, metastasis, sentinel lymph node, sentinel lymph node biopsy, sentinel node, sentinel node biopsy, vulva cancer, vulvar cancer, breast cancer	Different technical surgical approaches of groin lymph nodes
Cluster 5	groin, lymph node, postoperative complications, radiation, vulvar and vaginal carcinoma, vulvar diseases, vulvar lichen sclerosus, vulvar neoplasm, vulvar neoplasms	Radiation and complication of groin lymph node management
Cluster 6	local recurrence, lymph node metastasis, prognostic factors, recurrence, staging, survival	Evaluation of prognosis (recurrence, survival)
Cluster 7	anal cancer, genital warts, penile cancer	Biologically similar tumors/diseases

The list of the ten most highly cited manuscripts on VC (see Table 4) is headed by a meta-analysis by De Vuyst et al., published in 2009 in the *International Journal of Cancer* (Wiley). To date, it has accrued 819 citations.

**Table 4:** The ten most cited articles relevant to VC research (sorted in descending order)

Authors	Title	Source title (publisher)	Citations	Year	Citation density
De Vuyst et al. [16]	Prevalence and type distribution of human papillomavirus in carcinoma and intraepithelial neoplasia of the vulva, vagina and anus: A meta-analysis	International Journal of Cancer (Wiley)	819	2009	51.2
Van der Zee et al. [17]	Sentinel node dissection is safe in the treatment of early-stage vulvar cancer	Journal of Clinical Oncology (American Society of Clinical Oncology )	636	2008	37.4
Huh et al. [18]	Final efficacy, immunogenicity, and safety analyses of a nine-valent human papillomavirus vaccine in women aged 16–26 years: a randomised, double-blind trial	Lancet (Elsevier)	405	2017	50.6
Naumann et al. [19]	Safety and Efficacy of Nivolumab Monotherapy in Recurrent or Metastatic Cervical, Vaginal, or Vulvar Carcinoma: Results From the Phase I/II CheckMate 358 Trial	Journal of Clinical Oncology (American Society of Clinical Oncology )	390	2019	65
Judson et al. [20]	Trends in the incidence of invasive and in situ vulvar carcinoma	Obstetrics And Gynecology (Lippincott Williams & Wilkins)	367	2006	19.3
Kosary et al. [21]	FIGO stage, histology, histologic grade, age and race as prognostic factors in determining survival for cancers of the female gynecological system: an analysis of 1973-87 SEER cases of cancers of the endometrium, cervix, ovary, vulva, and vagina	Seminars in Surgical Oncology (Wiley-Liss)	361	1994	11.6
Lee et al. [22]	Long-term Management of Adult Vulvar Lichen Sclerosus A Prospective Cohort Study of 507 Women	JAMA Dermatology (American medical Association)	339	2015	33.9
Beesley et al. [23]	Lymphedema after gynecological cancer treatment - Prevalence, correlates, and supportive care needs	Cancer (Wiley)	324	2007	18
Levenback et al. [24]	Lymphatic Mapping and Sentinel Lymph Node Biopsy in Women With Squamous Cell Carcinoma of the Vulva: A Gynecologic Oncology Group Study	Journal of Clinical Oncology (Lippincott Williams & Wilkins)	321	2012	24.7
de Sanjosé et al. [25]	Worldwide human papillomavirus genotype attribution in over 2000 cases of intraepithelial and invasive lesions of the vulva	European Journal of Cancer (Elsevier)	318	2013	26.5

## Discussion

Spanning more than 75 years of VC research our bibliometric analysis emphasizes several major insights, including the following: (a) The observed publication trends reveal a marked acceleration in output, particularly over the past two decades, reflecting a significant shift in which VC has gained broader visibility and recognition within the academic community. (b) *Gynecologic Oncology* and *International Journal Of Gynecological Cancer* are by far the most prominent scientific journals publishing research on VC – a finding that is generally compatible with similar studies previously published [26]. (c) In terms of publication volume, the United States is by far the leading contributor; – however, such geographical differences are likely shaped by variations in research priorities, funding structures, and the strength of local research infrastructure.

Interestingly, considering only the ten most frequently used keywords as initial cluster analysis the keywords did not appear to yield meaningful thematic structures. Only after performing a subsequent and more detailed analysis comprising all seven individual sub-clusters, distinct thematic groupings (conceptual themes: therapy and treatment, epidemiology, tumor biology/pathology/diagnosis, different technical surgical approaches of groin lymph nodes, radiation and complication of groin lymph node management, evaluation of prognosis (recurrence, survival), biologically similar tumors/diseases - refer to Table 3) could be identified. Since these clusters encompass research domains ranging from tumor epidemiology and pathobiology to treatment strategies and prognostic evaluation, they reflect the thematic diversity and multiple scientific dimensions of this field. Furthermore, they also highlighted that knowledge advancement, even within this highly specialized subfield of gynecological oncology, relies on an interdisciplinary research effort encompassing clinical, translational, and basic science expertise.

The examination of the most highly cited studies provided a clearer understanding of the research landscape, in which sentinel lymph node dissection and HPV type distribution (the most cited study by De Vuyst et al. examined HPV prevalence, genotype distribution, and associated clinicopathological characteristics in precursor lesions and neoplasms of the vulva, vagina, and anus) emerged as recurrent themes [16, 17, 24, 25]. From a clinical perspective, issues related to lymph node assessment and systemic treatment of recurrent/metastatic disease are particularly relevant. Notably, several aspects identified in our most highly cited studies, such as a minimum depth of invasion of 1 mm [24] or tumor size < 4 cm [17], are now reflected in international guidelines [2, 27]. Notably, a recent phase I/II clinical trial (*CheckMate 358*) investigating immunotherapy with nivolumab already appears among the most cited publications [19]—reflecting the growing prominence of immune checkpoint inhibitors in recent years as an adjunct to conventional chemotherapy in VC [28–30]. That said, the *NCCN CLINICAL PRACTICE GUIDELINES IN ONCOLOGY Vulvar Cancer Version 3.2024* already recommended nivolumab as one potential second-line treatment for HPV-associated recurrent/advanced VC, citing indeed the *CheckMate 358* trial [31]. Equally significant is the study addressing lymphedema, which highlights a high incidence in patients with VC, a finding already well recognized by clinicians and nurses working in this field [23].

Future research in VC has the potential not only to individualize patient treatment through molecular diagnostics and the identification of optimal biomarkers but also to advance therapy by evaluating and integrating antibody‑drug conjugates (ADCs) as targeted treatments, representing a potential milestone beyond surgery and conventional chemoradiation. Ultimately, these clinical efforts should culminate in prospective, multicenter trials to support the development of evidence-based guidelines and standardized care pathways.

This report has several limitations that should be acknowledged. As with any bibliometric analysis, the findings are constrained by the characteristics and indexing practices of the underlying database. In this study, we selected WoS due to its inherent advantages, including superior citation indexing and broader coverage compared with databases such as PubMed or Scopus [14]. Moreover, recently published papers of substantial relevance may be underrepresented when assessed solely through citation counts (bias due to temporal influences); to mitigate this limitation, citation density was also reported. Although the authors sought to maintain maximal objectivity, identifying the most highly cited works on vulvar neoplasms required the exclusion of basic science articles from this sub-analysis, introducing a small but notable risk of subjective bias. Finally, citation bias, such as the preferential citation of certain journals or colleagues, cannot be entirely ruled out; however, quantification of publication volume and citation counts is not necessarily indicative of superior scientific quality or clinical relevance.

To maintain a comprehensive and unbiased bibliometric approach, our search strategy did not impose any restrictions on publication type, and the same principle was applied to the non-English literature. Although conference abstracts or non-English literature are often excluded during the search in bibliometric studies, we considered conference abstracts to represent a meaningful scientific contribution, as they provide an important platform, particularly for early career researchers, to disseminate preliminary or emerging findings, engage in scholarly exchange, and foster dialogue with peers. Such interactions may serve as catalysts for future studies and the initiation of international collaborations.

In conclusion, to our knowledge, this study presents the largest bibliometric analysis of VC to date, a topic that is gaining increasing attention in gynecological research, as reflected in the temporal trends observed in our analysis. We identified and visualized the most influential geographic regions in VC research, highlighted the leading journals, and presented the most highly cited publications, providing a comprehensive overview of research trends and hotspots. Accordingly, this study may serve as a practical resource for researchers, for instance, when selecting the most relevant journals for their VC studies.

## Supplementary Information

Below is the link to the electronic supplementary material.

Supplementary Material 1.

## Data Availability

The datasets generated and/or analyzed during the current study are available from the corresponding author upon reasonable request.
