# Supplementary material for: A bibliometric analysis of the research landscape of vulvar cancer
Source: Discov Oncol. 2026 Feb 17;17:361. doi: 10.1007/s12672-026-04573-5 (PMC12929766; doi:10.1007/s12672-026-04573-5)
Supplement: Supplementary file 1 — Supplementary Material 1. [file 12672_2026_4573_MOESM1_ESM.docx]

**SUPPLEMENTARY MATERIAL** - A Bibliometric Analysis of the Research Landscape of Vulvar Cancer


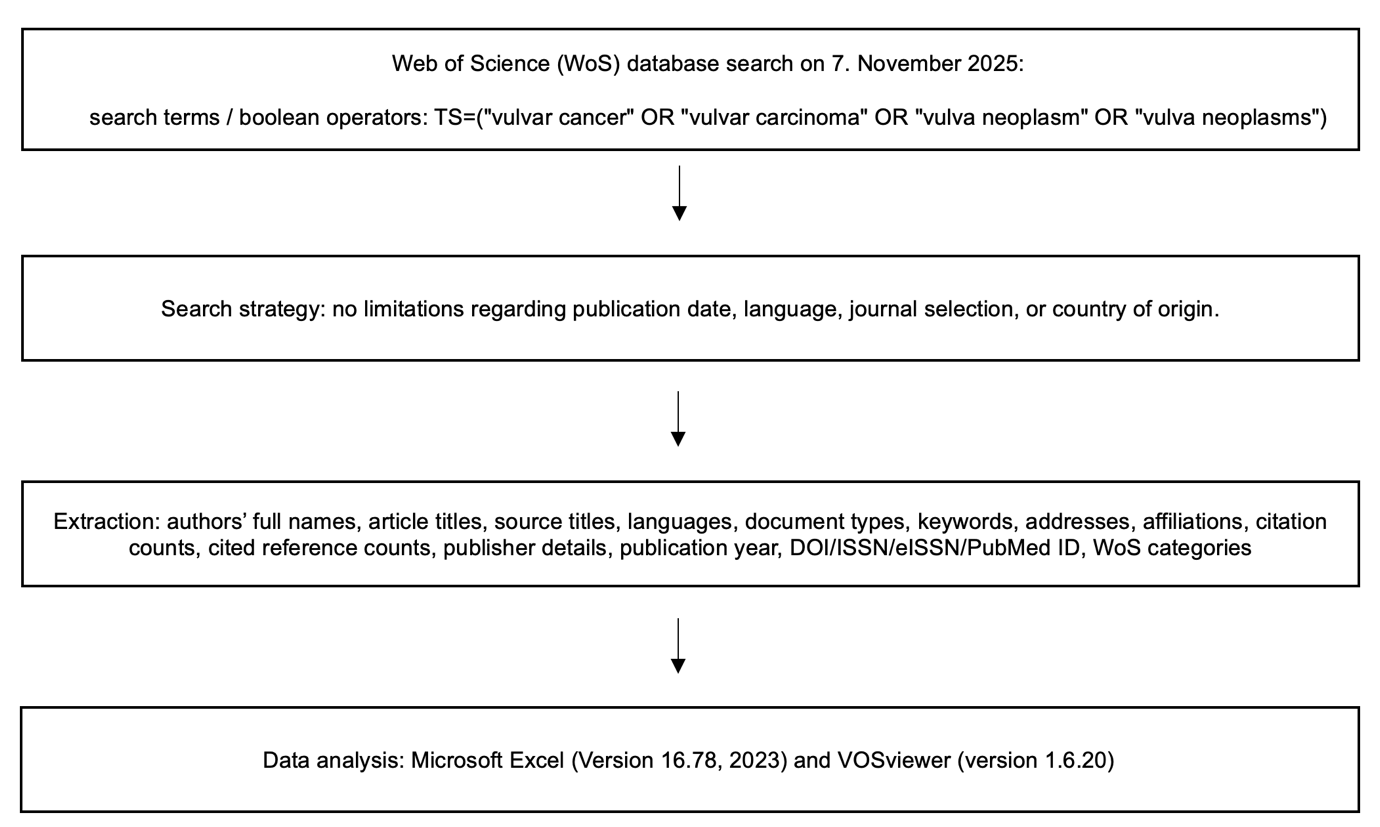


**Supp. Figure 1.** Flow diagram illustrating the methodology used for literature searching and data extraction.

**Supp. Figure 2.** Annual scholarly output of original work in VC research from 1946 to 2025. X-axis: years; y-axis: number of publications.

**Supp. Figure 3**. Number of citations per year in the field of VC research. -axis: years; y-axis: number of citations.

| **Document Type** | **Number of publications** |
| --- | --- |
| Article | 2218 |
| Meeting Abstract | 672 |
| Review | 451 |
| Editorial Material | 113 |
| Article; Proceedings Paper | 83 |
| Letter | 76 |
| Note | 15 |
| Correction | 9 |
| Proceedings Paper | 9 |
| Article; Early Access | 8 |
| Article; Book Chapter | 7 |
| News Item | 4 |
| Editorial Material; Early Access | 2 |
| Review; Early Access | 2 |
| Article; Retracted Publication | 2 |
| Book Review | 1 |
| Retraction | 1 |

**Supp. Table 1.** Distribution of article types

| **Organization** | **Publications** | **Citations** | **Average citations per article** |
| --- | --- | --- | --- |
| Univ Cattolica Sacro Cuore | 85 | 1156 | 13,6 |
| Radboud Univ Nijmegen | 65 | 2672 | 41,1076923 |
| Leiden Univ | 64 | 3099 | 48,421875 |
| Univ Med Ctr Hamburg Eppendorf | 62 | 1174 | 18,9354839 |
| Univ Texas Md Anderson Canc Ctr | 61 | 1302 | 21,3442623 |
| Fdn Policlin Univ A Gemelli Irccs | 55 | 536 | 9,74545455 |
| Univ Groningen | 53 | 2616 | 49,3584906 |
| Univ Toronto | 52 | 1675 | 32,2115385 |
| Mem Sloan Kettering Canc Ctr | 50 | 1557 | 31,14 |
| Univ Pittsburgh | 46 | 946 | 20,5652174 |

**Supp. Table 2.** Publication outcome according to the respective academic institution.

| **Keyword** | **Occurrences** |
| --- | --- |
| Vulvar Cancer | 1141 |
| Vulvar Carcinoma | 234 |
| Cervical Cancer | 194 |
| Vulva | 127 |
| Hpv | 119 |
| Human Papillomavirus | 118 |
| Radiotherapy | 111 |
| Prognosis | 109 |
| Squamous Cell Carcinoma | 109 |
| Vulvar Neoplasms | 107 |

**Supp. Table 3.** Most common keywords identified via keyword analysis.
